# Physician-Visit Frequency and Its Impact on Glycemic Control in People With Type 2 Diabetes: Quantifying Care Acceptance Parameters From Retrospective Electronic Health Record Data

**DOI:** 10.7759/cureus.76527

**Published:** 2024-12-28

**Authors:** Mukulesh Gupta, Tanusree Gupta, Tuhina Gupta

**Affiliations:** 1 General Medicine, Udyaan Health Care Pvt. Ltd., Lucknow, IND; 2 Obstetrics and Gynecology, Udyaan Health Care Pvt. Ltd., Lucknow, IND; 3 Obstetrics and Gynecology, Shree Guru Gobind Singh Tricentenary University Medical College, Hospital and Research Institute, Gurugram, IND

**Keywords:** doctor-patient rapport, electronic patient records, hemoglobin a1c (hba1c), patient compliance, standardized delta hba1c (δhba1c), type 2 diabetes, visit per quarter ratio (vqr), medication adherence

## Abstract

Objective: Type 2 diabetes is a metabolic disorder characterized by insulin resistance and hyperglycemia affecting many individuals worldwide. For effective management, adherence to recommended physician visits is important, along with lifestyle modification and pharmacological interventions. Regular doctor visits can improve adherence and help prevent complications. This study examined how doctor-visit frequency impacts blood glucose (BG) control in people with type 2 diabetes.

Research design and methods: This retrospective, single-center study included adults with type 2 diabetes who had at least two visits and two hemoglobin A1c (HbA1c) values recorded at least 90 days apart between March 2019 and November 2023. Visit per quarter ratio (VQR) and the standardized delta HbA1c (ΔHbA1c) were defined. Data were analyzed for descriptive statistics and compared for significance between the groups. Statistical differences among the groups were determined using the Kruskal-Wallis test and Dwass-Steel-Critchlow-Fligner pairwise comparisons.

Results: Five hundred seventy-seven participants with type 2 diabetes were analyzed, with a mean age of 53.9 years (±12.7). The HbA1c outcome was significantly lower in the good compliance group (7.3%) than in the poor (7.7%). The high VQR group had a significantly lower HbA1c outcome (7.4%) than the low VQR group (7.7%); 50.98% (n = 104) in the low VQR group, 65.54% (n = 97) in the medium VQR group, and 60.44% (n = 136) in the high VQR group achieved HbA1c below the target. The mean ΔHbA1c was significantly lower in the good compliance group compared to the poor compliance group. The average follow-up durations were 11.22 quarters (±4.11) for low VQR, 9.75 quarters (±4.69) for medium VQR, and 5.50 quarters (±4.84) for high VQR.

Conclusion: A higher frequency of follow-up may be needed to encourage people with type 2 diabetes mellitus to visit their doctor regularly. The frequency of doctor visits has a positive impact on BG control. Regular visits enable timely adjustment of therapy and ensure high compliance with prescribed treatment. These findings have significant implications for mitigating nonadherence in chronic conditions like type 2 diabetes mellitus and warrant further investigation.

## Introduction

Type 2 diabetes, a chronic disorder triggered by unhealthy lifestyles, managed by pharmacotherapy and possibly reversible with comprehensive care, is rapidly spreading in India and globally. The Centers for Disease Control and Prevention labeled it an epidemic in 1994 and, in 2005, called it a "diabetes pandemic" [1]. Global prevalence has tripled to 537 million adults in two decades, projected to reach 783 million by 2045. India has about 74.2 million (77 million as per the World Health Organization) people living with diabetes, ranking second globally [2].

Despite advancements in evidence, technology, and pharmacology, type 2 diabetes outcomes remain suboptimal. The LongitudinAl Nationwide stuDy on Management And Real-world outComes of diabetes in India trial showed only 20% of participants achieved hemoglobin A1c (HbA1c) <7% [3]. Improved screening methods, artificial intelligence-enabled testing, and new drug classes with fewer side effects and additional benefits have emerged. However, the limited impact on outcomes suggests investigating other factors that are not yet mainstream in research.

While clinical care and patient education are crucial for outcomes, "care acceptance" by patients also plays a significant role [4]. Factors like regular follow-ups, medication compliance, active lifestyle, and dietary adherence can significantly influence outcomes, highlighting gaps in comprehensive care.

A systematic review by Xu et al. highlighted the critical role of physician-patient encounters in managing type 2 diabetes, including medication tracking, self-care guidance, health monitoring, and early prevention/treatment of complications [5]. The American Diabetes Association (ADA) 2022 guideline recommends assessing glycemic status at least twice yearly for patients meeting goals and quarterly for those with recent therapy changes or unmet goals [6]. The National Institute for Health and Care Excellence guidelines suggest measuring HbA1c every three to six months until stable [7].

Adherence to these recommendations is pivotal; a lack of regular engagement with the physician and his team has been reported to impact clinical outcomes negatively. This retrospective analysis of the diabetes care hospital electronic health care records (EHR) was undertaken to analyze the impact of physician-visit frequency and outcomes in HbA1c in a real-world single-center setting. The primary research question was whether maintaining optimum physician-visit frequency helps achieve glycemic targets (HbA1c, 7.5%) and helps in improving glycemic control in people with type 2 diabetes: Delta HbA1c (ΔHbA1c) = baseline HbA1c - outcome HbA1c. The paper highlights a strategy for measuring and documenting visit compliance among people with type 2 diabetes who are actively seeking clinical care in a real-world setting by removing the effect of treatment duration and allowing for comparison between different patients and their visit-compliance behavior. The study also aims to evaluate the correlation between physician-visit frequency and HbA1c outcomes in patients with type 2 diabetes, highlighting the impact of regular follow-ups on achieving better glycemic control. The approaches identified in this paper are simple, quantifiable, and useful for an unbiased evaluation of one of the most important factors in the "care acceptance" dimension in a clinical setting.

## Materials and methods

Design

**Figure 1:**
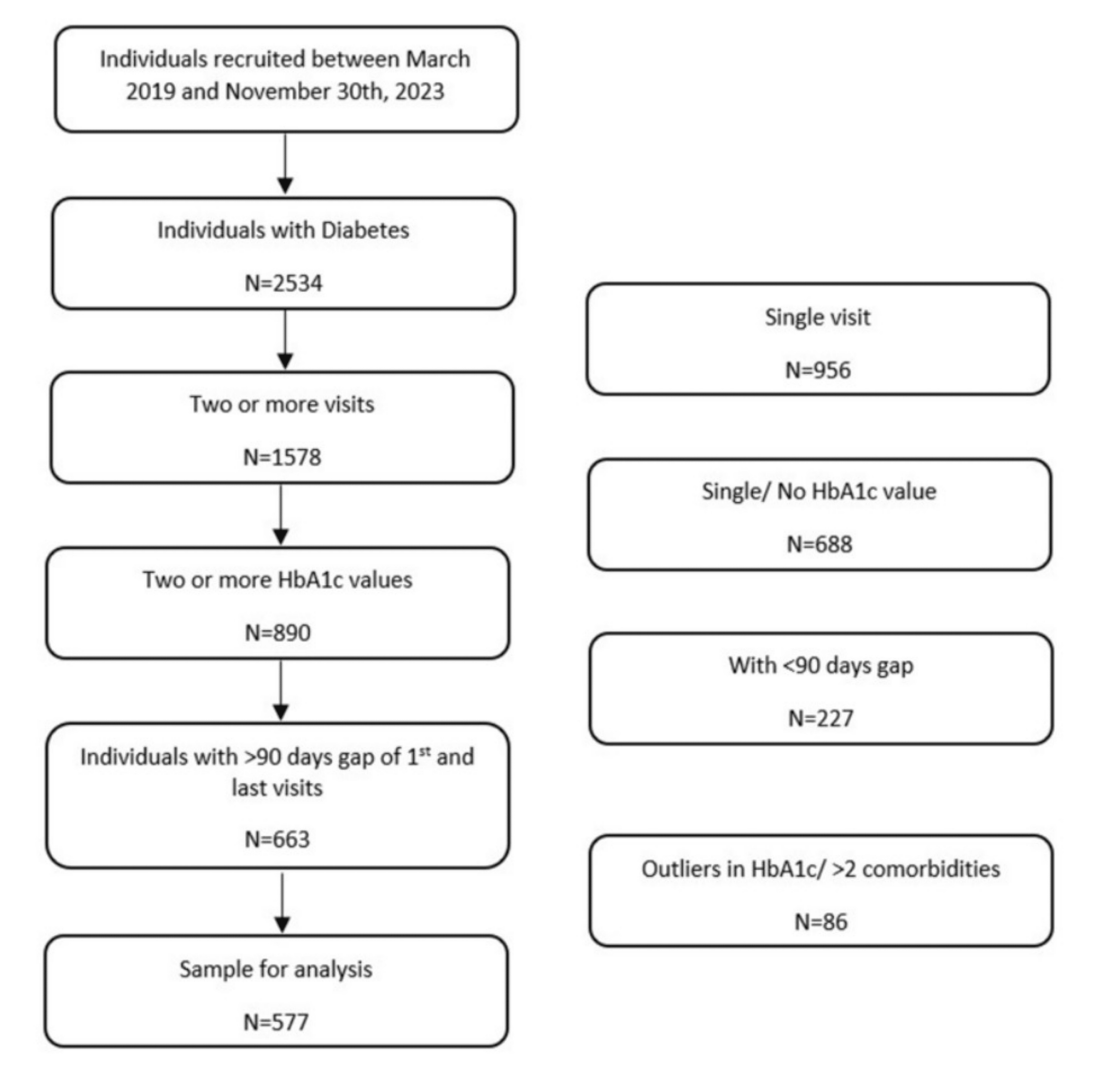
Patient flow HbA1c: hemoglobin A1c

This was a retrospective, single-center study in which participant data were extracted from the EHR. Inclusion criteria included adult participants diagnosed with type 2 diabetes who had at least two visits and two HbA1c values separated by 90 days between March 2019 and November 30, 2023. They should also have two or fewer comorbidities, including congestive artery disease, asthma, chronic kidney disease, chronic liver disease, chronic obstructive pulmonary disorder, and hypertension. In the present study, all patients were provided standard care per clinical guidelines. This included lifestyle modification advice and management with approved antihyperglycemic therapies tailored to their individual glycemic status (Figure 1).

Data source and extraction

Data from Medeva EHR were extracted programmatically using the inclusion criteria. Patient identifiers were excluded, and confidentiality was maintained throughout. Variables included age, gender, diabetes duration, visit dates, HbA1c values (Logical Observation Identifiers Names and Codes), type 2 diabetes diagnosis, and comorbidities (Systematized Nomenclature of Medicine-Clinical Terms). The patient disposition process included 2,534 people with diabetes, of which 890 had two or more visits and HbA1c values. Among them, 663 had more than 90 days between their first and last visits. After excluding those with more than two comorbidities, the final sample for analysis was 577 participants.

Ethical considerations

Participants signed informed consent forms at registration, allowing the use of anonymized data for research. The study followed the ethical guidelines of the Indian Council of Medical Research. The Udyaan Healthcare Institutional Review Board approved the study on July 25, 2023 (Q-CAP/MG/23).

Definitions and variables

Visit per Quarter Ratio

The total number of visits and the total duration of treatment (calculated as the number of quarters) were calculated for each patient from the data. These two variables' ratios were calculated to generate the visit per quarter ratio (VQR; the number of visits divided by the total number of quarters completed by the patient under care).

Standardized Delta HbA1c (Standardized ΔHbA1c)

The difference between the initial HbA1c measurement and the latest available HbA1c value was calculated for each patient (ΔHbA1c: baseline HbA1c value - recent HbA1c value). This value was divided by the total duration of the participant’s treatment, which was measured in the number of quarters.

Study groups

Participants with a VQR > 1 (i.e., on average, they visited the clinician at least once per quarter or more) were classified as "good compliance," and participants with VQR < 1 (i.e., on average, they did not visit the clinician at least once per quarter) were classified as "poor compliance" to generate compliance-based groups. Additionally, VQR was used to create three groups: low (<0.75 visits per quarter), medium VQR (0.75 and 1.25 visits per quarter), and high VQR (>1.25 visits per quarter) to generate VQR-based groups. Glycemic outcomes were described in three formats: mean outcome HbA1c for different groups, target HbA1c (7.5%) achievement, and standardized ΔHbA1c.

Statistical analysis

Data were cleaned, and the study variables and groups were calculated from the data described in the previous section. Descriptive tables and visual graphs were generated to understand data patterns and trends. Based on normality tests, parametric or nonparametric tests were employed to test different hypotheses. Compliance-based groups (good or bad compliance) were evaluated for differences in 1) mean outcome HbA1c, 2) target HbA1c (7.5%) achievement, and 3) standardized ΔHbA1c. Similarly, the VQR-based groups (high, medium, and low) were also evaluated for differences in 1) mean outcome HbA1c, 2) target HbA1c (7.5%) achievement, and 3) standardized ΔHbA1c. Although the target HbA1c achievement of 7.5% has been described earlier and is the preference of the authors of this paper, it has also been reported at 7.0% and 8.0%. Thus, while reporting the target HbA1c achievement for the different groups, every 0.5% HbA1c step change was included in the graphs to allow widespread generalizability of the results. Additionally, the change in HbA1c has been visually reported in greater further detail by considering six categories ranging from ≥(+)1%, (+)0.5-0.9%, (+)0.0-0.4%, (-)0.1-0.4%, (-)0.5-0.9%, and ≥(-)1%. The data from the center were explored using Python 3.6 (Python Software Foundation, Wilmington, DE) for initial understanding and overview. Multiple visualizations were created to understand the data structure and quality better. A planned statistical analysis was performed using Statistical Package for the Social Sciences version 18; SPSS Inc., Chicago, IL; and Jamovi; jamovi (Version 2.3) (Computer Software). Retrieved from https://www.jamovi.org. The data were also analyzed for descriptive statistics like mean, median, mode, and standard deviation and compared for significance between patient groups on various parameters. The statistical difference among the groups was observed using the Kruskal-Wallis test, followed by pairwise comparisons with the Dwass-Steel-Critchlow-Fligner test.

## Results

A total of 2,534 participants with a diagnosis of type 2 diabetes were registered in the EHR at the time of data extraction for this study. The mean age of the cohort was 53.9 years (±12.7), with a gender distribution of 1,149 female participants (45.35%) and 1,385 male participants (54.65%), and an average body mass index (BMI) of 27.6 (±5.0) kg/m². From this initial cohort, 577 participants met the inclusion criteria and were included in the statistical analysis. This subgroup had a mean age of 56.1 years (±11.6), a gender distribution of 274 female participants (47.49%) and 303 male participants (52.51%), and an average BMI of 27.6 (±4.7) kg/m². Demographic details of the sample can be found in Table 1. The mean baseline HbA1c of the total sample (n = 577) was 8.52% (±2.28), and the mean HbA1c outcome was 7.5% (±1.70), with a mean change of 0.85% (±2.13).

**Table 1:** Demographic profile of total sample and sample used for statistical analysis after exclusion as per methods SD: standard deviation; BMI: body mass index; CVDs: cardiovascular disorders

Characteristics	Total sample profile	Participants selected in the final analysis
n	2,534	577
Age (mean ± SD), years	53.9 ± 12.7	56.1 ± 11.6
Gender, n (%)		
Female	1,149 (45.35%)	274 (47.49%)
Male	1,385 (54.65%)	303 (52.51%)
BMI (mean ± SD), kg/m^2^	27.6 ± 5.0	27.6 ± 4.7
Baseline HbA1c (mean ± SD), %	8.5 ± 2.3	8.4 ± 2.1
Number of visits (mean ± SD)	3.6 ± 4.5	8.6 ± 6.5
Duration of diabetes in years	7.8 ± 7.0	7.9 ± 7.1
CVD, n (%)	17 (0.7%)	11 (1.9%)
Hypertension, n (%)	1,172 (46.3%)	314 (54.4%)
Dyslipidemia, n (%)	28 (1.1%)	16 (2.8%)
Obesity, n (%)	76 (3%)	44 (7.6%)
Other comorbidities, n (%)	79 (3.1%)	65 (11.3%)

Mean HbA1c outcomes

Participants with poor compliance (n = 284) with a baseline HbA1c of 8.1% (±1.93) achieved an outcome of 7.7% (±1.77) with a mean change of 0.435 (±1.89), and participants with good compliance (n = 293) with a baseline HbA1c of 8.6% (±2.25) achieved an HbA1c outcome of 7.3% (±1.61) with a mean change of 1.26% (2.27). The HbA1c outcome in good compliance was significantly lower than the outcome in participants with poor compliance (nonnormal data distribution, p value of 0.009 using Mann-Whitney U test). Participants with low VQR (n = 284) with a baseline HbA1c of 8.1% (±1.96) achieved an outcome of 7.7% (±1.80) with a mean change of 0.42 (±1.83), participants with medium VQR (n = 148) with a baseline HbA1c of 8.2% (±1.97) achieved an outcome of 7.4% (±1.54) with a mean change of 0.80 (±2.19), and participants with high VQR (n = 225) with a baseline HbA1c of 8.6% (±2.30) achieved an outcome of 7.4% (±1.69) with a mean change of 1.28 (±1.83). The HbA1c outcome in the high VQR group was significantly lower than the outcome in low VQR (nonnormal data distribution, statistical difference between three groups was significant with a p value of 0.0104 using Kruskal-Wallis test, followed by pairwise comparison using Dwass-Steel-Critchlow-Fligner pairwise comparison test high VQR vs. low VQR, p value 0.049; high VQR vs. medium VQR, p value 0.787; and low VQR vs. medium VQR, p value 0.237).

Target HbA1c (7.5%) outcomes

A total of 58.41% of the sample (n = 337) achieved an HbA1c value below the target threshold of 7.5%. In the poor compliance group, 54.58% (n = 155) were below the target HbA1c value of 7.5%, while 62.12% (n = 182) of the good compliance group participants were below the target value. This difference was statistically significant (p value of 0.066 using χ² test). In the low VQR group, 50.98% (n = 104) of the participants were below the target HbA1c value, 65.54% (n = 97) of those in the medium VQR group were below the target HbA1c value, and participants with high VQR 60.44% (n = 136) were below the target value. These differences were significantly significant (statistical difference between the three groups was statistically significant with a p value of 0.017 using the test, followed by pairwise comparison using χ² test: high VQR vs. low VQR, p value 0.017; high VQR vs. medium VQR, p value 0.320; and low VQR vs. medium VQR, p value 0.006) (Tables 2-4).

**Table 2:** Mean HbA1c outcomes comparison between poor compliance and good compliance p = 0.066 using χ² test between mean HbA1c outcomes comparison vs. compliance HbA1c: hemoglobin A1c

Compliance	<7.5% of HbA1c, n (%)	≥7.5% of HbA1c, n (%)
Poor compliance	155 (54.58%)	129 (45.42%)
Good compliance	182 (62.12%)	111 (37.88%)

**Table 3:** Mean HbA1c outcomes comparison between low, medium, and high VQR groups p = 0.040 using χ² test between mean HbA1c outcomes vs. VQR groups HbA1c: hemoglobin A1c; VQR: visit per quarter ratio

Mean HbA1c	Low VQR (<0.75), n (%)	Medium VQR (0.75-1.25), n (%)	High VQR (>1.25), n (%)
≤5.9%	23 (11.27%)	18 (12.16%)	41 (18.22%)
6-6.4%	17 (8.33%)	15 (10.14%)	23 (10.22%)
6.5-6.9%	34 (16.67%)	31 (20.95%)	43 (19.11%)
7-7.4%	30 (14.71%)	33 (22.3%)	29 (12.89%)
7.5-7.9%	32 (15.69%)	11 (7.43%)	29 (12.89%)
8-8.4%	22 (10.78%)	12 (8.11%)	21 (9.33%)
8.5-8.9%	14 (6.86%)	4 (2.7%)	13 (5.78%)
9-9.4%	6 (2.94%)	5 (3.38%)	7 (3.11%)
9.5-9.9%	2 (0.98%)	7 (4.73%)	2 (0.89%)
≥10%	24 (11.76%)	12 (8.11%)	17 (7.56%)

**Table 4:** Mean HbA1c outcomes comparison between six categories of VQR p = 0.102 using χ² test between mean HbA1c outcomes vs. six categories of VQR groups HbA1c: hemoglobin A1c; VQR: visit per quarter ratio

Mean HbA1c	<0.50 VQR, n (%)	0.50-0.74 VQR, n (%)	0.75-0.99 VQR, n (%)	1-1.24 VQR, n (%)	1.25-1.50 VQR, n (%)	>1.50 VQR, n (%)
≤5.9%	11 (10.58%)	12 (12%)	10 (12.5%)	8 (11.76%)	12 (25%)	29 (16.38%)
6-6.4%	10 (9.62%)	7 (7%)	7 (8.75%)	8 (11.76%)	2 (4.17%)	21 (11.86%)
6.5-6.9%	15 (14.42%)	19 (19%)	14 (17.5%)	17 (25%)	4 (8.33%)	39 (22.03%)
7-7.4%	14 (13.46%)	16 (16%)	20 (25%)	13 (19.12%)	8 (16.67%)	21 (11.86%)
7.5-7.9%	14 (13.46%)	18 (18%)	4 (5%)	7 (10.29%)	7 (14.58%)	22 (12.43%)
8-8.4%	10 (9.62%)	12 (12%)	6 (7.5%)	6 (8.82%)	5 (10.42%)	16 (9.04%)
8.5-8.9%	8 (7.69%)	6 (6%)	3 (3.75%)	1 (1.47%)	4 (8.33%)	9 (5.08%)
9-9.4%	3 (2.88%)	3 (3%)	4 (5%)	1 (1.47%)	2 (4.17%)	5 (2.82%)
9-9.9%	1 (0.96%)	1 (1%)	3 (3.75%)	4 (5.88%)	0 (0%)	2 (1.13%)
≥10%	18 (17.31%)	6 (6%)	9 (11.25%)	3 (4.41%)	4 (8.33%)	13 (7.34%)

ΔHbA1c

A total of 61.53% (n = 355) of the sample demonstrated no change or improvement in their HbA1c values when compared to the baseline measurements. In the poor compliance group, 56.34% (n = 160) of the participants underwent no change or improvement, whereas 66.55% (n = 195) of participants in the good compliance group underwent no change or improvement in their HbA1c values compared to baseline. The mean ΔHbA1c in the good compliance was significantly lower than the poor compliance group (nonnormal data distribution, with a p value of <0.001 using the Mann-Whitney U test). In the low VQR group, 56.37% (n = 115) experienced no change or improvement in their HbA1c values, while in the medium VQR group, 61.49% (n = 91) participants underwent no change or improvement in their HbA1c values, and among participants in the high VQR group, 66.22% (n = 149) experienced no change or improvement in their HbA1c values. These differences were also statistically highly significant (nonnormal data distribution, statistical difference between three groups was significant with a p value of 0.005 using Kruskall-Wallis test, followed by pairwise comparison using Dwass-Steel-Critchlow-Fligner pairwise comparison test high VQR vs. low VQR, p value 0.004; high VQR vs. medium VQR, p value 0.176; and low VQR vs. medium VQR, p value 0.509) (Tables 5-7).

**Table 5:** ΔHbA1c outcomes comparison between poor compliance and good compliance p = 0.012 using χ² test between ΔHbA1c outcomes vs. compliance HbA1c: hemoglobin A1c

Compliance	No change or improvement, n (%)	Deterioration, n (%)
Poor compliance	160 (56.34%)	124 (43.66%)
Good compliance	195 (66.55%)	98 (33.45%)

**Table 6:** ΔHbA1c outcomes comparison between low, medium, and high VQR groups p = 0.627 using χ² test between ΔHbA1c outcomes vs. VQR groups HbA1c: hemoglobin A1c; VQR: visit per quarter ratio

ΔHbA1c	Low VQR (<0.75), n (%)	Medium VQR (0.75-1.25), n (%)	High VQR (>1.25), n (%)
≥(+)1%	72 (35.29%)	59 (39.86%)	102 (45.33%)
(+)0.5-0.9%	21 (10.29%)	15 (10.14%)	26 (11.56%)
(+)0.0-0.4%	22 (10.78%)	17 (11.49%)	21 (9.33%)
(-)0.4-0.1%	33 (16.18%)	15 (10.14%)	25 (11.11%)
(-)0.9-0.5%	22 (10.78%)	16 (10.81%)	21 (9.33%)
≥(-)1%	34 (16.67%)	26 (17.57%)	30 (13.33%)

**Table 7:** ΔHbA1c outcomes comparison between six categories of VQR p = 0.750 using χ² test between ΔHbA1c outcomes vs. six categories of VQR groups HbA1c: hemoglobin A1c; VQR: visit per quarter ratio

ΔHbA1c	<0.50 VQR, n (%)	0.50-0.74 VQR, n (%)	0.75-0.99 VQR, n (%)	1-1.24 VQR, n (%)	1.25-1.50 VQR, n (%)	>1.50 VQR, n (%)
≥(+)1%	37 (35.58%)	35 (35.00%)	30 (37.50%)	29 (42.65%)	20 (41.67%)	82 (46.33%)
(+)0.5-0.9%	13 (12.50%)	8 (8.00%)	6 (7.50%)	9 (13.24%)	5 (10.42%)	21 (11.86%)
(+)0.1-0.4%	10 (9.62%)	12 (12%)	9 (11.25%)	8 (11.76%)	3 (6.25%)	18 (10.17%)
(-)0.4-0.1%	19 (18.27%)	14 (14.00%)	7 (8.75%)	8 (11.76%)	5 (10.42%)	20 (11.30%)
(-)0.9-0.5%	13 (12.50%)	9 (9.00%)	12 (15.00%)	4 (5.88%)	6 (12.50%)	15 (8.47%)
≥(-)1%	12 (11.54%)	22 (22.00%)	16 (20.00%)	10 (14.71%)	9 (18.75%)	21 (11.86%)

Rationale for standardized ΔHbA1c: differences in treatment duration

Mean follow-up durations (number of quarters) for the total sample (n = 577), participants with poor compliance, and participants with good compliance were 8.61 quarters (±5.22), 11.04 quarters (±4.25), and 6.26 quarters (±4.99), respectively. The difference in the treatment duration for the poor compliance group was statistically highly significantly greater than that of the good compliance group (nonnormal data distribution, p value of <0.001 using Mann-Whitney U test). Additionally, the average durations of follow-up for low VQR, medium VQR, and high VQR were 11.22 quarters (±4.11), 9.75 quarters (±4.69), and 5.50 quarters (±4.84), respectively. These differences were also statistically highly significant (nonnormal data distribution, statistical difference between three groups was highly significant with a p value of <0.001 using Kruskall-Wallis test, followed by pairwise comparison using Dwass-Steel-Critchlow-Fligner pairwise comparison test high VQR vs. low VQR, p value <0.001; high VQR vs. medium VQR, p value <0.001; and low VQR vs. medium VQR, p value 0.005). Standardized ΔHbA1c was described to remove the effect of treatment duration as a confounder.

Standardized ΔHbA1c

Percentage distributions of no change or improvement and deterioration using standardized ΔHbA1c are the same as those reported using ΔHbA1c previously. There was a statistically significant difference in the mean standardized ΔHbA1c of the good compliance group compared with the poor compliance group (nonnormal data distribution, with a p value of <0.001 using the Mann-Whitney U test). The differences in mean standardized ΔHbA1c between low, medium, and high VQR were also statistically highly significant (nonnormal data distribution, statistical difference between three groups was significant with a p value of <0.001 using Kruskall-Wallis test, followed by pairwise comparison using Dwass-Steel-Critchlow-Fligner pairwise comparison test high VQR vs. low VQR, p value <0.001; high VQR vs. medium VQR, p value 0.009; and low VQR vs. medium VQR, p value 0.311).

When comparing the proportion of patients who experienced no change or improvement in the good compliance and poor compliance groups, there was a statistically significant difference, with a greater proportion of participants in the good compliance group experiencing some form of improvement or no change compared to their baseline HbA1c values (p value of 0.012 using χ² test). This difference in the proportions of participants experiencing no change or improvement was statistically different between the high VQR and low VQR groups (p value of 0.036 using χ² test) and not statistically different between the high VQR and medium VQR (p value of 0.350 using χ² test), and medium VQR and low VQR (p value of 0.336 using χ² test) (Tables 8-10).

**Table 8:** Standardized ΔHbA1c outcomes comparison between poor compliance and good compliance p = 0.012 using χ² test between standardized ΔHbA1c outcomes vs. compliance HbA1c: hemoglobin A1c

Compliance	No change or improvement, n (%)	Deterioration, n (%)
Poor compliance	160 (56.34%)	124 (43.66%)
Good compliance	195 (66.55%)	98 (33.45%)

**Table 9:** Standardized ΔHbA1c outcomes comparison between low, medium, and high VQR groups p < 0.001 using χ² test between standardized ΔHbA1c outcomes vs. VQR groups HbA1c: hemoglobin A1c; VQR: visit per quarter ratio

Standardized ΔHbA1c	Low VQR (<0.75), n (%)	Medium VQR (0.75-1.25), n (%)	High VQR (>1.25), n (%)
≥(+)1%	94 (46.08%)	61 (41.22%)	64 (28.44%)
(+)0.5-0.9%	7 (3.43%)	13 (8.78%)	34 (15.11%)
(+)0.0-0.4%	14 (6.86%)	17 (11.49%)	51 (22.67%)
(-)0.4-0.1%	13 (6.37%)	15 (10.14%)	43 (19.11%)
(-)0.9-0.5%	14 (6.86%)	13 (8.78%)	25 (11.11%)
≥(-)1%	62 (30.39%)	29 (19.59%)	8 (3.56%)

**Table 10:** Standardized ΔHbA1c outcome comparison between six categories of VQR p < 0.001 using χ² test between standardized ΔHbA1c outcomes vs. six categories of VQR groups HbA1c: hemoglobin A1c; VQR: visit per quarter ratio

Standardized ΔHbA1c	<0.50 VQR, n (%)	0.50-0.74 VQR, n (%)	0.75-0.99 VQR, n (%)	1-1.24 VQR, n (%)	1.25-1.50 VQR, n (%)	>1.50 VQR, n (%)
≥(+)1%	53 (50.96%)	41 (41.00%)	33 (41.25%)	28 (41.18%)	19 (39.58%)	45 (25.42%)
(+)0.5-0.9%	3 (2.88%)	4 (4.00%)	4 (5.00%)	9 (13.24%)	5 (10.42%)	29 (16.38%)
(+)0.0-0.4%	4 (3.85%)	10 (10.00%)	8 (10.00%)	9 (13.24%)	4 (8.33%)	47 (26.55%)
(-)0.4-0.1%	5 (4.81%)	8 (8.00%)	7 (8.75%)	8 (11.76%)	7 (14.58%)	36 (20.34%)
(-)0.9-0.5%	6 (5.77%)	8 (8.00%)	9 (11.25%)	4 (5.88%)	7 (14.58%)	18 (10.17%)
≥(-)1%	33 (31.73%)	29 (29.00%)	19 (23.75%)	10 (14.71%)	6 (12.5%)	2 (1.13%)

## Discussion

This study analyzed 2,534 participants diagnosed with type 2 diabetes from EHR. The mean age of participants was 53.9 years, with a slight female predominance (45.35%) and a mean BMI of 27.6 kg/m². A detailed analysis was conducted on 577 participants who met the inclusion criteria, and female participants were significantly underrepresented (10.79%). Gender differences in healthcare utilization have been studied extensively, and numerous papers have identified this as an important barrier to sufficient self-care [8-11].

Numerous guidelines recommend individualizing care plans with follow-up visits every six or three months. A systematic review by Xu et al. reports numerous studies where physician visits every three months have been shown to improve HbA1c outcomes and reduce the number of complications [5]. VQR is being defined explicitly for the first time in this paper, considering the extensive but nonsystematic review of the literature done for this manuscript. This paper has also introduced the concept of standardizing the HbA1c change across the treatment duration for patients using standardized delta HbA1c.

To meet the alarming rise in and challenging demands in the treatment of metabolic disorders in China, the National Metabolic Management Center (MMC) was founded in 2016. These centers have advanced medical equipment and Internet of Things technology to facilitate better online and offline integrated care for patients with diabetes and other metabolic disorders. Zhao et al. studied whether the follow-up frequency of people with type 2 diabetes leads to different clinical outcomes [4]. A total of 19,908 participants with at least six months of facility-based follow-up were recruited in the MMCs between June 2017 and April 2021 and divided into lower frequency (LFF, <2.0 per year) and higher frequency follow-up (HFF, >2.0 per year) groups. They created multivariable linear regression models to assess the relationship between follow-up frequency and between-group glycemic percentage changes (fasting blood glucose, BG; and HbA1c). They reported that participants with HFF showed a significantly greater decrease in percentage changes of fasting BG (4.95% ± 37.96% vs. 2.21% ± 43.08%, p < 0.0001) and HbA1c (12.14% ± 19.78% vs. 9.67% ± 20.29%, p < 0.0001) after adjustments compared to those with LFF [4].

Another retrospective study by Luo et al. studied whether follow-up frequency impacts metabolic control in people with type 2 diabetes by exploring the relationship between clinical follow-up frequency and target-reaching rates for HbA1c, low-density lipoprotein (LDL), and blood pressure [12]. Participants were divided into three groups according to follow-up frequency: <4 times/year, =4 times/year, and >4 times/year. The authors reported that positive changes in HbA1c and LDL were associated with follow-up frequency and that, after adjusting for confounders, the target-reaching rate of HbA1c for patients with follow-up ≥4 times/year was higher than those with <4 times/year, with odds ratios of 1.518 (=4 times/year) and 1.508 (>4 times/year), respectively. Compared to patients with follow-up <4 times/year, the target-reaching rate of LDL for patients with follow-up ≥4 times/year was higher than those with =4 times/year, with odds ratios of 1.998 (=4 times/year) and 2.517 (>4 times/year).

Numerous randomized controlled trials (RCTs) have been done earlier to study the impact of physician visits on clinical outcomes in type 2 diabetes. Wermeling et al. studied 2,215 patients over an 18-month follow-up duration with two groups with monitoring frequency of three or six months and reported that 93.3% of the participants in the three-month follow-up frequency achieved an HbA1c target of 7.%% compared to 92.0% of the participants in the six-month frequency group [13]. Another RCT by Hu et al. studied 155 participants with type 2 diabetes in two groups with follow-up frequency of either monthly or three-monthly and reported that the mean HbA1c outcome in the monthly follow-up group was 5.04% (±1.15) compared to 6.25% (±0.58) in the three-monthly group [14].

A study from the United States by Ye et al. studied the number of physical encounters in a six-month period in 2,124 participants with type 2 diabetes and reported that one additional physical encounter in six months marginally increased the probability of transition from a diabetic state to a prediabetic state (BG >125 mg/dL) by 4.3%; and from a prediabetic state (BG 100-125 mg/dL) to nondiabetic state (BG <100 mg/dL) by 3.2% [15].

Therapeutic inertia in people with type 2 diabetes has been studied extensively across diverse aspects of treatment in the literature. Failure to intensify therapy (or deintensify therapy), when indicated, has been defined as therapeutic inertia by multiple sources [16-18]. Inability to intensify treatment because of patient-behavior factors has not been studied in detail. McDaniel et al. reported that HbA1c values were higher among patients exposed to therapeutic inertia during the first six months of treatment initiation [19]. Tsai et al. [18] studied the effect of mandatory monthly outpatient visits on therapeutic inertia in patients with suboptimal control of type 2 diabetes and reported that monthly outpatient visits could improve therapeutic inertia in patients with poorly controlled type 2 diabetes. The ADA, in collaboration with the American College of Physicians and the National Diabetes Education Program, recently published a Quality Improvement Success Story by Oshman et al. [20]. The project identified 411 patients with poor glycemic control, of which 82 met the inclusion criteria and were eligible to accept the diabetes-focused-visiting (DFV) scheme. Of the 29 patients who accepted the DFV, the average baseline HbA1c was 9.2%, and the average outcome HbA1c reported was 8.5%, with an average improvement of -0.9%. Thirty-five patients receiving usual care had an average baseline HbA1c of 9.6% and an average improvement in HbA1c of -0.1%.

While treatment intensification and individualization of treatment strategy are critical in improving clinical outcomes in people with type 2 diabetes, it can be argued that unless patients show up for follow-up visits and maintain a steady visit frequency as advised by the physician, any form of clinical outcome will remain left to chance. This remark, although empirical, can now be supported by evidence from this paper. Significant deterioration (standardized ΔHbA1c of ≥ -1%) reduces as the VQR increases, as shown in Table 9. However, more importantly, significant improvement (standardized ΔHbA1c of ≥+1%), as well as significant deterioration (standardized ΔHbA1c of ≥ -1%), occurs in almost comparable proportions in participants with a low VQR (<0.75 visits per quarter), as shown in Tables 9, 10.

While improving clinical outcomes remains the primary objective of any clinical practice guideline, preventing deterioration or delay in complications also remains an integral pillar for the clinician. Similar to the findings of Luo et al., >4 times/year does not offer any additional advantage for the glycemic target or the rate of reaching glycemic targets. Our findings also suggest that high VQR does not lead to better HbA1c target outcomes [12]. However, when reviewing the change in HbA1c, our study reports that participants who had a high VQR had a higher number of participants with any (high, medium, and low) improvement and a lower number of participants with any deterioration in their standardized delta HbA1c compared to those with low or medium VQR (Tables 8-10).

This paper provides several important insights into the relationship between physician-visit frequency and glycemic outcomes in patients with type 2 diabetes. It demonstrates that standardized ΔHbA1c can serve as a valuable metric for comparing HbA1c changes across studies involving participants with varying treatment durations, particularly in real-world settings. The VQR can be used as a metric in research and clinical practice to categorize patients based on their historic visit-frequency behavior. The low or poor VQR is associated with poorer mean HbA1c outcomes, poorer achievement of target HbA1c goals, and poorer changes in HbA1c compared to baseline. Conversely, a medium or optimal VQR drives better HbA1c outcomes and targets HbA1c achievement compared to low or poor VQR, but high or excessive VQR does not offer any additional benefit. VQR greater than medium or optimal drives positive change in HbA1c improvement, wherein higher VQR can be associated with greater HbA1c improvement.

The study's strengths are a large sample size, real-world data from EHR, and the VQR use, which allows for a more nuanced analysis of how visit frequency impacts HbA1c outcomes. Using standardized ΔHbA1c, which accounts for differences in treatment duration, provides a robust measure of glycemic control improvement. This standardization helps mitigate potential biases related to varying follow-up durations among participants. The retrospective nature of this study inherently limits the ability to establish causality. The analysis did not account for factors such as medication adherence, lifestyle changes, duration of diabetes, socioeconomic status, and access to healthcare services. Future research could incorporate these variables to provide a more comprehensive analysis.

## Conclusions

The present study demonstrates a clear correlation between physician-visit frequency and HbA1c outcomes in patients with type 2 diabetes. Participants who adhered to regular quarterly visits exhibited significantly better glycemic control than those with fewer visits. Specifically, the good compliance group showed a greater reduction in HbA1c levels, higher achievement rates of target HbA1c values, and a higher proportion of participants with no change or improvement in HbA1c compared to the poor compliance group. Similarly, the high VQR group had significantly better glycemic outcomes than the low and medium groups. These findings underscore the importance of consistent and frequent clinical follow-ups in managing type 2 diabetes. Regular visits facilitate timely adjustments to treatment plans, close monitoring of glycemic levels, and prompt interventions collectively contribute to improved diabetes management. The results also highlight that routine care, without the necessity of frequent special care visits due to comorbidities, is sufficient to achieve improvements in glycemic outcomes.

In conclusion, the present study provides strong evidence that frequent physician visits are crucial for optimal glycemic control in patients with type 2 diabetes. These findings support the need for healthcare policies and practices that encourage regular follow-ups to enhance diabetes management and improve long-term patient outcomes.
